# Long-Term Potentiodynamic Testing and Tribometric Properties of Amorphous Alloy Coatings under Saline Environment

**DOI:** 10.3390/molecules27041421

**Published:** 2022-02-19

**Authors:** Amjad Iqbal Falak, Ayesha Iqbal, Grzegorz Moskal, Muhammad Yasir, Abdullah I. Al-Mansour, Mohammad Amir Khan, Shamshad Alam, Muhammad Shahbaz, Adeel Zia, Ahsan Ejaz

**Affiliations:** 1 Department of Advance Materials & Technologies, Faculty of Materials Engineering, Silesian University of Technology, 44-100 Gliwice, Poland; grzegorz.moskal@polsl.pl; 2 Centre of Industrial Policy and Market Analysis, Faculty of Quality and Industrial system Engineering, University of Punjab, Lahore 54000, Pakistan; ayeshafalak565@gmail.com; 3 Department of Materials Science & Engineering, Institute of Space Technology, Islamabad 44000, Pakistan; muhammadyasir85@gmail.com; 4 Department of Civil Engineering, College of Engineering, King Saud University, Riyadh 11421, Saudi Arabia; amansour@ksu.edu.sa (A.I.A.-M.); salam@ksu.edu.sa (S.A.); 5 Department of Civil Engineering, Galgotias College of Engineering and Technology, Greater Noida 201310, India; amir.khan@galgotiacollege.edu; 6 Department of Organic Chemistry, Faculty of Chemistry and Pharmacy, Ludwig Maximilian University of Munich, 81377 Munich, Germany; mushch@cup.uni-muenchen.de; 7 School of Chemistry and Chemical Engineering, Shanghai Jiaotong University, Shanghai 200240, China; adeel101@sjtu.edu.cn; 8 Department of Civil Engineering, Mirpur University of Science and Technology, Mirpur10250, Pakistan; ahsanejaz.ce@must.edu.pk

**Keywords:** amorphous alloys, Fe-based amorphous coating, atmospheric plasma spray, amorphicity, corrosion resistance, passive layers, wear resistance

## Abstract

Protective coatings for harsh environments are always welcome, but they must overcome profound challenges, including corrosion and wear resistance. The purpose of this study is to look into the long-term potentiodynamic polarization measurements and dry tribometric behavior of plasma-sprayed amorphous coatings on AISI 1035 mild steel. To investigate the impact of unique active polarization potentials on the electrochemical studies of the iron-based amorphous layer, which compares favorably to AISI 1035 mild steel, the active potential polarization curve and friction coefficient tests were performed. Scanning electron microscopy (SEM) and energy dispersive x-ray (EDX) analyses were used to investigate the coating’s corrosion behavior. Their mechanical (Tribometric tests at higher sliding speeds) and chemical properties (electrochemical potentiodynamic polarization investigations) have also been thoroughly investigated. There is enough validation that these protective coatings can be used in hostile environments. The effects of long-term corrosion for 24 and 48 h were thoroughly examined. Tribometric examinations revealed that amorphous layers are highly resistant under dry conditions, as they offered a very low and stable friction coefficient less than 4 μ with micro Vickers hardness 1140 ± 22.14 HV, which is more than twice as compared to mild steel AISI 1035. The corrosion resistance of coatings in 3.5 wt % NaCl solution displays active transition characteristics of activation, passivation, over passivation, and pitting, as shown by the potentiodynamic polarization curves.

## 1. Introduction

The iron-based amorphous coatings originated from the iron-based bulk metallic glasses (Fe-BMGs). Fe-BMGs are well-known for their excellent intensive properties such as soft magnetic behavior, low thermal conductivity, outstanding mechanical properties, and high corrosion resistance. The thermal spraying approach [1,2,3,4,5] allows for the fabrication of these coatings over a large area with no size restrictions.

These innovative coatings can be manufactured in various ways. The critical factor for forming amorphous coatings is the rapid condensation of powder on the surface, which forms various meta-stable amorphous contents. The instant condensation of powder particles on the substrate depends on the employed thermal spray techniques because of their varied speeds and cooling rates [6,7]. The amorphous coating, namely the lack of crystalline phases, dislocations, and crystal defects, rapidly develops passive films on their surfaces. Several amorphous coatings (Ni-based, Co-based, Fe-based, Ti-based, Al-based, Mg-based, Cu-based) have been developed, which are highly corrosion resistant. The most prominent amorphous system is Fe-based, especially alloys containing yttrium (Y), because of their outstanding mechanical and chemical properties.

The limitations include poor impact resistance and fracture toughness. Literature shows this limitation can be overcome and made twice as strong by reinforcing carbon amorphous phases and adding a NiCrAlY layer as a bond coat (BC) [8].

Recently, it was investigated that Fe-based amorphous coatings exhibit long-term corrosion-resistant like surface exposure to 5 wt % NaCl neutral salt spray tests for 15 days with no rust and peeling observed on the surface [9]. It was reported that FeMoCrYCB amorphous coating would be a potential candidate to protect 921A navy steel, stainless steel 304, Cr plated alloy and Ti-6Al-4V alloy in aggressive sulfuric acid solution [10,11]. Wang et al. proposed that increasing the chromium content in the alloying elements increases the intensive corrosion property but affects the amorphicity of coatings. It was also predicted that molybdenum favours the formation of amorphous phase’s greatly and affects thermal stability [12].

An iron-based amorphous coatings attributed to low porosity and high hardness exhibits excellent wear-resistant properties [13]. When employed for long-term service, they exhibit degradation processes like these: oxidative wear [14], abrasive wear [15] and fatigue wear [16]. Zhang et al. [17] discussed the wear behaviour of the amorphous coated layer and concluded that the wear rate depends on the sliding speed of the indenter, not on the load provided to the coatings. They have applications in human joint replacements [18], the mining industry like drill bits under abrasive conditions, anti-adhesion material [19], high-tech fields like telecommunication, and in the military due to their soft magnetic property and high strength [20]. Iron-based advanced coatings also finds their employment in defence applications like nuclear fuel containers and naval shipping in the marine environment. These types of improved materials can be applied for various purposes, including transportation vehicles. The global push for green powder energy and the formation of cheap electricity from nuclear technologies necessitate some robust coatings must be developed for aggressive environment [21].

According to the report presented by the National Association of Corrosion Engineers (NACE) Islamabad Section, an under-developed country like Pakistan can save 270–280 billion Pakistani rupees annually through corrosion mitigation in the local industrial sectors [22]. AISI 1035 is a commonly used steel grade in industries, because of its high strength, low cost, and environmental friendliness, medium carbon steel; however, there are severe limitations, such as greater susceptibility to corrosion and rust [23]. Iron-based amorphous coatings are considered as high-performance corrosion resistant materials (HPCR) that can mitigate the corrosion-issues in industries [24]. The novelty of this article is that we developed a low-cost coatings (mono-layer) by using low-cost carbon steel, so the commercialization of these coatings can be achieved for local industries. Secondly, we focused on the long-term electrochemical corrosion tests for SAM1651 under potentiodynamic polarization measurements and investigated the wear properties at a higher rate. In the present article, we are using the alloy FeMoCrYCB for the fabrication of the coatings and emphasizing this alloy due to its high-performance corrosion-resistant property in an aggressive environment. Secondly, due to the low manufacturing cost of these coatings, we can diminish the corrosion concerns of local industries, especially for the oil and gas industries. Hence, from the above literature, it is concluded that Fe-based amorphous coatings are high-performance corrosion-resistant coatings (HPCR), but their extended term-potentiodynamic corrosion resistance property was not previously discussed. This article presents the investigation on the monolithic amorphous layer and the comparison of previous results in the literature, with the novelty that long-immersion in the saline environment and high speed for wear rate, that are briefly discussed, it expands coatings applications in new emerging fields.

## 2. Results and Discussion

### 2.1. Microstructure and Characterization Analysis

**Figure 1:**
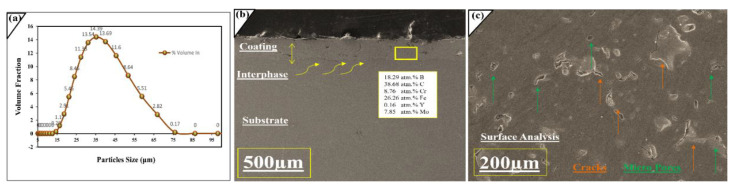
(**a**) Measurement of particles size distribution (**b**) Cross-section and interface of Amorphous coated sample (**c**) Surface analysis of coatings.

**Figure 2:**
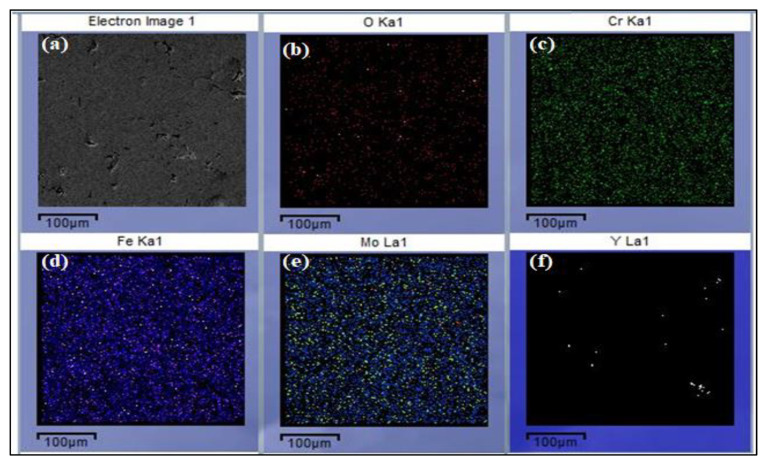
Morphology and Elemental Maps from Coated Surface including the (**a**–**f**) bullets.

The cross-sectional analysis of the fabricated coating was observed via SEM. The measured thickness of the coating is 200 μm. Figure 1b shows the cross-sectional area of the coated layer, and atomic percentages were investigated in the fabricated layer via energy dispersive X-ray analysis (EDX). It can be seen that the amorphous layer was successfully prepared and remains compact with the substrate due to a strong interlocking mechanical bonding effect. It also reveals the interface layer between the coating and the substrate. This layer has SiO_2_ particles, which generate roughness. The interface layer indicates that a good mechanical and metallurgical bond has developed between both the substrate and molten particles. Zhu et al. proposed that the formation of nanoscale SiO_2_ prevents the formation of interfacial imperfections and embrittlement issues and enables the coating to provide good fracture toughness [25]. Therefore, sandblasting was performed prior to the thermal spray process to achieve optimum surface roughness. As it can be seen at 200 μm Figure 1c, the effect of porosity is there. It developed due to the bombardment of semi-melted particles on the surface. ImageJ Software reveals the average level of porosity is 2.0%, which was calculated by SEM surface image analysis. Figure 2a was obtained from the surface of the mono-layer coatings, and maps were drawn based on compositions of feedstock alloy, which indicates the distribution of major alloying elements like iron, chromium, and molybdenum is relatively homogeneous and uniform on the surface.

### 2.2. Phase Composition of the Coatings

**Figure 3:**
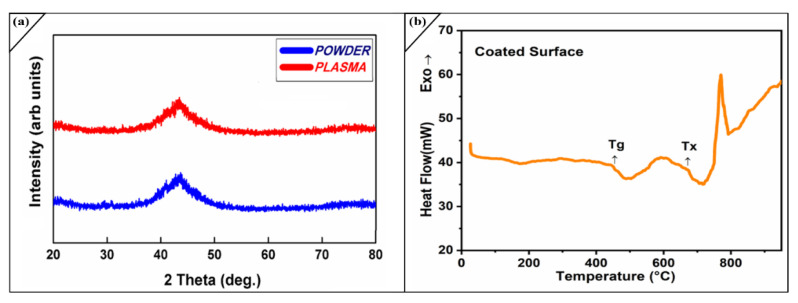
(**a**) XRD configurations (Amorphous coating) and Powder sample (**b**) DSC obtained pattern as-sprayed (Fe-based coating).

X-ray diffraction is the most commonly used practice for identifying non-crystalline phases. XRD was performed with CuKα radiation. Figure 3a shows the amorphous behaviour of powder and coating; it can be observed that the XRD pattern analysis confirmed the absence of crystalline phases. There is a sharp, broad hump obtained between 25° and 55° = 2θ for the powder and coatings, which reveals its amorphous nature, remains intact after passing the powder through the hot flame of APS. This is also confirmed by SEM, where no grain boundaries appear, and non-crystalline phases are confirmed in our fabrication. Our previous studies reveal that an APS coating with 2.5% porosity gives approximately 80% amorphous content [26].

The calculation was done by using Equation (1).

(1) $$x_{\mathrm{am.C}}=\Delta h_{C}/\left(\Delta h_{\mathrm{C.C}}\right)$$

X_am,C_ represents the amorphous content of the coating, Δh_C_ the crystallization energy of the coating and Δh_c.c_ the crystallization energy of the corroded coating. The reason for this analysis, is due to strong attack of harsh environment on the oxide layer and bottom layer of coating. The high salty environment initiated pitting corrosion and disturbed the distribution of elements in the monolithic amorphous layer; this allows the formation of various other oxides and developing crystalline phases. We have also observed this is due to decrease in the thickness of coated layer. On the surface of coatings some un-melted particles are compressed by the high velocity of employed thermal spray technique. As the upper splat corroded under an aggressive environment, due to its high hardness, the lower un-melted splat also leaves the surface, making the porous surface more vulnerable for corrosion attacks. Figure 3b In the DSC curve of the coatings, it was revealed that amorphous system following multi-step crystallization process with the onset crystallization temperature (T_x_) of around 700 °C, while glass transition temperature (T_g_) for the amorphous layer starts 435 °C. It is also observed the higher value of T_x_ alloys had a larger glass-forming ability (GFA). There is some minor chemical reaction of oxide occurs due to impurities in the running system. We compared it by data provided for thermal gravimetric curve, which indicates no mass changes. The amorphous content in the APS coating was decreased after electrochemical corrosion.

### 2.3. Mechanical Studies of Coated Layer

**Figure 4:**
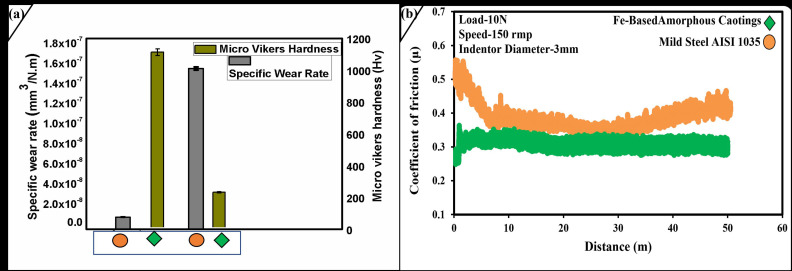
(**a**) Specific Wear Rate and Micro Vickers hardness upon polished surface on Amorphous layer and AISI 1035 (**b**) Coefficient of friction under dry sliding distance of 50 m for Amorphous Layer and AISI 1035.

Vickers microhardness measurements were further carried out on the surface of monolayer coatings, which yield a hardness of 1140 ± 22.14 HV for the coatings and 227 ± 4.5 HV for the AISI 1035 substrate, indicating that obtained layer is hard as compared to its soft substrate surface. These measurements are concluded based on an applied load of 10 N with indenter for 30 s. The obtained results are the mean of 5 indentations. 200 μm layer of amorphous coating when subject to perform bending testing under normal conditions, it was found that coating remains for 25 s before fracture appears on it. Further investigation reveals the fracture appears in the area, where the thickness of coating was primarily low and the porosity content was marked higher. Figure 4a reveals that the substrate micro-Vickers hardness is 5 folds less than the obtained hardness of amorphous coatings. In other word we can summarize this “eighty percent hardness increase by 200 μm of amorphous layer on AISI 1035”. In the reference of Archard’s equation, which states that higher hardness of amorphous layer aids the wear resistant property of amorphous coating. Figueroa et al. [27] attribute that boron has a greater effect in the hardness of amorphous layer and increase its content; the level can be increased but up to certain limits.

The specific wear rate mentioned in Table 1 reveals that the wear resistant property of AISI 1035 increased much greater times with amorphous layer. Figure 4b shows us that the coefficient of friction for the amorphous layer is much lower and more stable than its parent substrate. It very well may be seen that a consistent state coefficient of friction graph bend with the mean value of the coefficient of friction around 0.31 μ was acquired. The coefficient of friction remains stable during the whole test. At the same time, the substrate coefficient of friction starts from 0.55 μ, end around 0.50 μ and is highly unstable. The above outcomes propose that the amorphous layer shows an outstanding anti wear-friction property.

**Figure 5:**
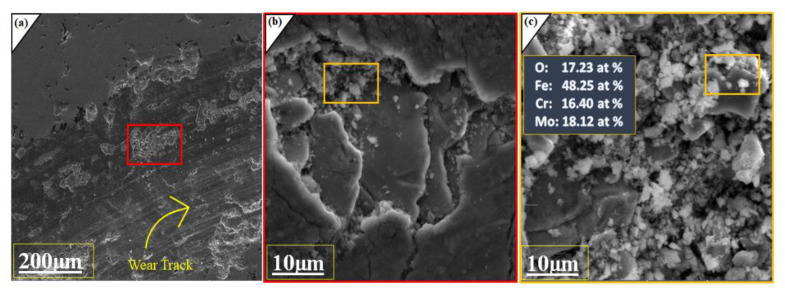
(**a**) SEM image of Wear track with 20.0 kv at 300X (**b**) intensifying SEM scan for wear-worn under loads (10 N) and dry, sliding velocity (150 rpm) 20.0 kv at 5.00 kx (**c**) SEM image of the wear debris at 20.0 kv at 5.00 kx and EDX analysis.

Furthermore, SEM images were taken to analyze the worn surfaces. Figure 5a,b were taken as the wear test done, and it was seen that the coatings layer remains intact with the substrate, but minor damages and cracks are visible in the SEM image. In the second part of the figure, some cracks are clearly visible on the surface due to high stresses and friction that appears during the experimentation. Figure 5b,c represents the intensifying coated SEM morphology of wear worn and wear-debris surface. The significant oxides produced during the wear test are Fe, Cr and Mo under dry sliding conditions. The calculated oxygen content was 17.23% after detections of Fe, Cr, Mo atomic percentage via Energy Dispersive X-ray Analysis (EDX) analysis on the worn surface, which provide enough proves that oxides layer which are adhesively combined with each other undergo wear. During the experimentation, the friction generated between the tip of indenter and surface leads to increased temperature and generates various oxides of Fe, Cr, and Mo. Iqbal et al. and xuqiang et al. support oxidative wear mechanism for iron-based amorphous coatings under dry sliding conditions [26,28]. It is generally observed that sliding speed of indenter increase the wear depth, that’s why we used 150 revolutions per minute speed from initially and remains constant until the 50 m of distance is covered by the indenter. This high speed generated the temperature, which aids the process of oxidation to major alloying elements. It was also observed that despite the adhesive wear oxidation tests, coatings is not peel off from the surface due to its higher micro Vickers hardness. Here it should be noted that porosity and amorphous content plays an important role; comparing investigated results with our previous work and literature reveals that low porosity level and higher amorphous content improve the anti-wear resistance property of the coatings [29].

### 2.4. Electrochemical Studies of Coated Layer

Potentiodynamic polarization measurements was performed to evaluate the anti-corrosion resistance of prepared iron-based amorphous coatings. As a fast and effective measuring method, the potentiodynamic polarization curve is the widely used to measure the potential, corrosion current density, and corrosion rate [30,31,32]. Electrochemical measurements were conducted open to air at room temperature using an electrochemical workstation (Reference 600^TM^) with a three-electrode system.

**Figure 6:**
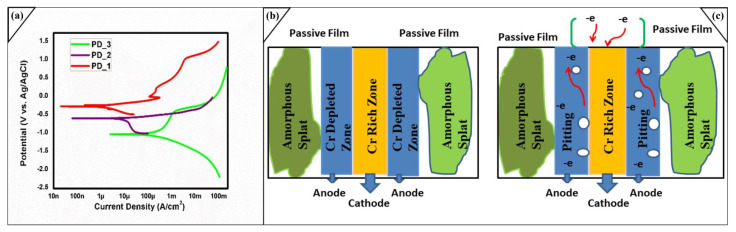
(**a**) Electrochemical studies of coating layer and bare substrate by using three electrode system. (**b**,**c**) A proposed graphical model for corrosion mechanism analysis.

**Figure 7:**
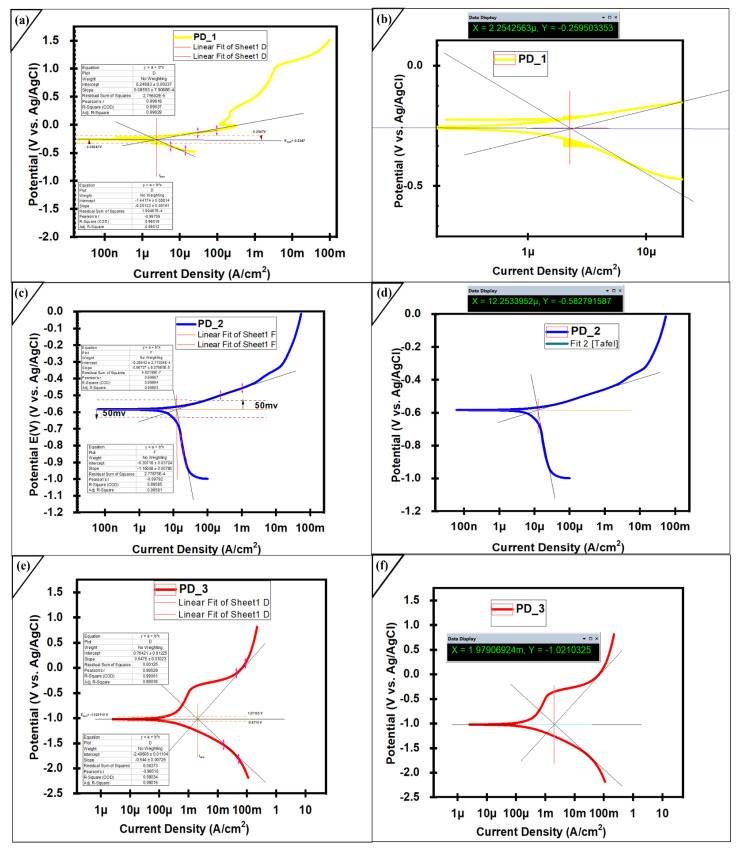
Electrochemical studies of coating layer (**a**) Tafel fit Potentiodynamic Polarization Curves PD_1 (**b**) Measurement of (Ecorr) and (I corr) for PD_1 (**c**) Tafel fit Potentiodynamic Polarization Curves PD_2 (**d**) Measurement of (Ecorr) and (I corr) for PD_2 layer (**e**) Tafel fit Potentiodynamic Polarization Curves PD_3 (**f**) Measurement of (Ecorr) and (I corr) for PD_3 layer. All the test were performed against Ag/AgCl reference electrode and Platinum as counter electrode. All measurements are done by using both anodic and cathodic region. In the extrapolation, it was insured that, all the extrapolation for anodic and cathodic region should start 50 mV away from the Ecorr. X = Current Density, Y = Corrosion Potential.

An electrolyte 3.5 wt % sodium chloride (by mixing 35.24 g of NaCl and 1000 mL distilled water) was prepared, the conductive polish surface was dipped in the solution for 24 h (PD_1). All the experimentations were carried out for amorphous coatings (−1.6 V to +1.6 V), and for substrate (−2.0 V to +2.0 V) applied potential. The scan speed of 1 mVs⁻¹ was used for substrate and coated samples. Potentiodynamic test was performed, and again, the same sample was soaked in 3.5% NaCl brine solution for another 24 h (PD_2). The polished and conductive mild steel AISI 1035 sample was soaked in the solution for 1 h. SEM and EDX analyzes were done to examine the degradation mechanism on the surface. It was revealed from Table 2 that the corrosion current density (I_corr_) is continuously increasing from PD_1 > PD_2 > PD_3. Figure 6a represents that various passive layers are developed that continuously protect the material from a corroded environment. The Passive region in PD_1 was broken once, but another stable passive oxide developed again. In case of PD_2 only one passive layer was present which is obtained after immersing the sample for 24 h, while in the case of PD_3 a steep passive layer which is effect of Fe_X_O_y_ obtained until the pitting start. PD_1 sample remains in 3.5 wt % solution for 24 h, while PD_2 sample was soaked for 48 h before Potentiodynamic test. It was investigated that despite of long term immersion in SAM1651, and least thickness in the manufactured coatings, its anti-corrosion property is quite comparable with the literature [33,34]. The electrochemical properties represented in Table 2 were obtained via Tafel fitting, which reveals that coating exhibits steepness in their passive layer at the range of ±50 mV potential related to the corrosion potential E_corr_. The order of E_corr_ values in Table 2 with respect to experiment is PD_1 > PD_2 > PD_3, while the corrosion current density (I_corr_) could be positioned in a contrary way like PD_1 < PD_2 < PD_3. In general, a smaller I_corr_ suggests a better corrosion resistance. Consequently, the obtained impressive corrosion-resistant values for the tests could be positioned as follows: PD_1 > PD_2 > PD_3. Figure 7b,d,f illustrate the determination of corrosion current density from the PD curves through anodic and cathodic regions for the coatings and substrate.

The corrosion rate of monolayer coating increased from 3.43 × 10^−6^ (mpy) to 9.25 × 10^−4^ (mpy). As we increased the time and saline environment concentration around the coated surface, it was observed corrosion rate increased. The sample PD_2 shows important anti-corrosion properties after immersion for 48 h in 3.5 wt % NaCl brine solution. Hence, impressive corrosion-resistant property for a long time with stability was achieved. The significant property of anti-corrosion for these coatings can be visualized that the amorphous coatings continually protects its substrate until all the elements get dissolved by the aggressive electrolyte environment.

The passive region of PD_1 is larger than PD_2 and PD_3, which predict the strong-firm passive regions, practically anti-corrosion property in a saline environment. Still, in the second scan Na^+^ ions and Cl^−^ ions make the ionic bonding and release the electropositive and electronegative ions from the surface. The surface contains micro-defects like micropores and flaws on the surface, within the coating layer, and on the interface that developed due to partial oxides and the solidification of semi-molten particles during thermal spraying. When the sample was dipped for long terms in the corroded environment, Cl- ions reached the microdefects and interrupted the passive film. The amorphous layer easily forms Cr_2_O_3_, as it contains Cr, Mo and Fe.

Furthermore, the presence of Mo indorsed the formation of the adhesive passive films and delayed the dissolution of Cr [35]. The removal of these ions develops the charges to its neighbours’ atoms, the porous areas are aiding this process and suppressing the passive layers continuously. The whole layer is split into two regions depleted zone and un-depleted zone. Figure 6b,c shows that corrosion mechanism initiates in the amorphous splats and various regions developed for chromium-rich and chromium depleted zones. Due to the electrochemical process, electrons are continually moving from anodic region to the cathodic region leaving behind the vacancy that later progresses into pitting. This behaviour shows that alloying elements oxidize from the anodic region, allowing the more aggressive Cl^−^ anion to attack additional elements. The exact process for molybdenum and iron appears until the metallic coatings becomes degraded and peeled off. In the whole system, brine solution acts as same seal level salt concentration and work as cathode while the fabricated-amorphous layer works as an anode. The oxidation at anode (metal gets oxidized) aid the corrosion process. So, to avoid corrosion degradation, minimum porosity level in the surface, minimum cracks and strong metallic bonding within coating is recommended. As it can be seen at 200 μm, the effect of porosity is there, which is the leading cause of degradation.

Figure 7 was obtained after applying Tafel Method to illustrate the current densities, corrosion potential, anodic and cathodic regions of the passive layers. While measuring current density, both anodic and cathodic curves were utilized. In the extrapolation, it was insured that, all the extrapolation for anodic and cathodic region should start 50 mV away from the Ecorr. In the Figure 7b,d,f, X shows Current Density and Y represents Corrosion Potential. Figure 7a,c,e illustrate about the measurement of anodic and cathodic beta from the data. Both the anodic and cathodic beta was calculated above and below 0.05 V references to Ecorr.

The corrosion rate was measured by employing the Equation (2),

Corrosion rate (mpy) = 0.13 × icorr × Ew/d(2)

where Ew is the equivalent weight, and d is density in g/cm^3^.

**Figure 8:**
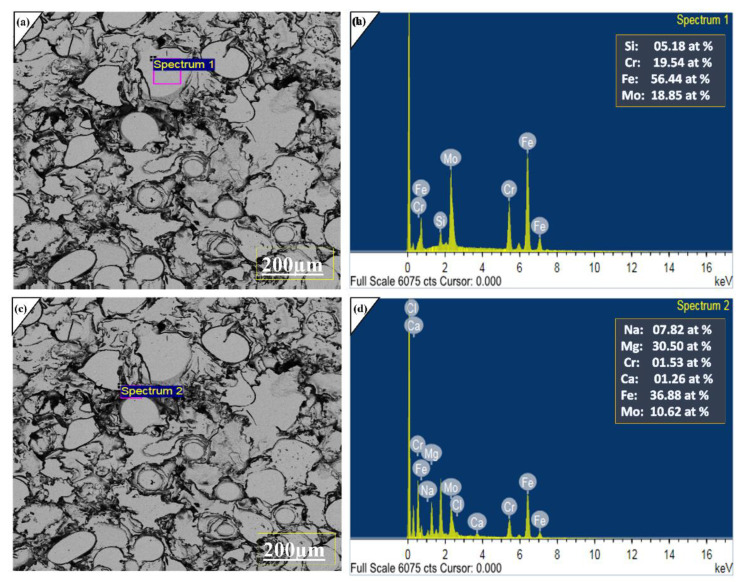
(**a**,**c**) SEM image of Corroded surface with 20.0 kv at 300X for Fe-based amorphous coatings under 3.5 wt % NaCl. (**b**,**d**) Represent EDX analysis of the corrosion product at two different spots. Processing option: All elements analyzed (Normalized).

Figure 8 was obtained from the corroded surface of the coating, EDX analysis reveals that the passive layer generally consist of (Fe_x_O_y_ + Mo_x_O_y_ + Cr_x_O_y_). The silicone particles at 200 μm indicate the amorphous layer was highly corroded and silicone travelled from the bottom layer. This is due to the interdiffusion effect which was speed up due to long term immersions. The effect of a salty environment reached up to its inner layer only where porosity is high, and thickness is low. Literature studies also reported that significant oxides of iron are FeO which convert into divalent Fe_2_O_3_ [36], Cr_2_O_3_ [37], MoO_2_ and MoO_3_ [38] obtained during the passivation layer and protect the substrate against corrosion attacks.

**Figure 9:**
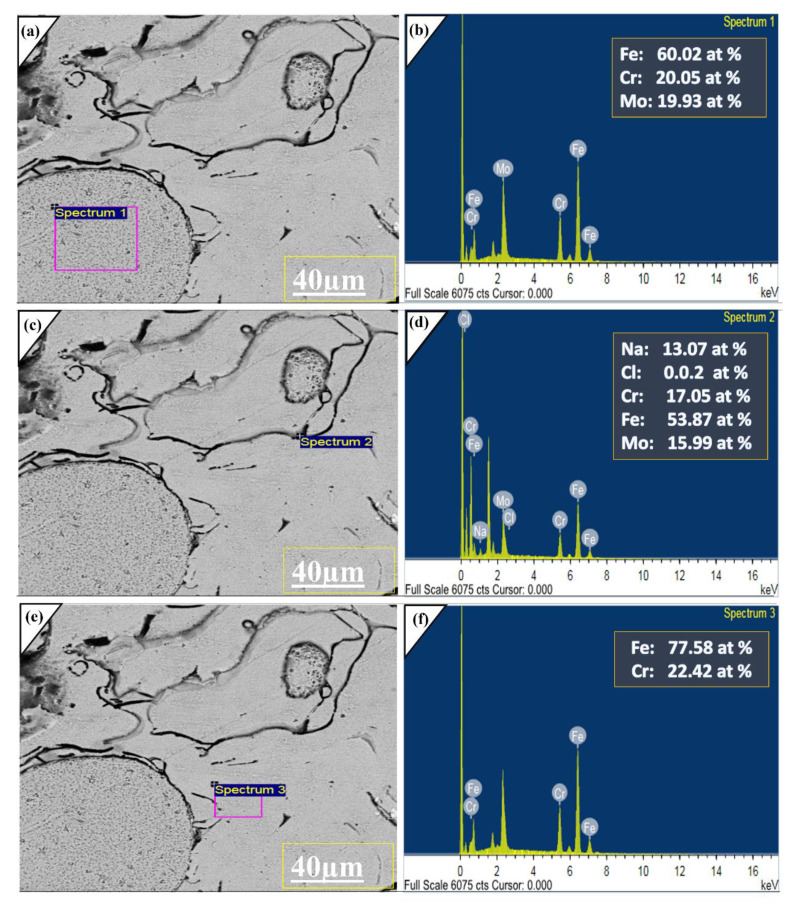
(**a**,**c**,**e**) SEM image of Corroded surface with 20.0 kv at 2.01 kX for Fe-based amorphous coatings under 3.5 wt % NaCl. (**b**,**d**,**f**) Represent EDX analysis of the corrosion product at the different spots. Processing option: All elements analyzed (Normalized).

To further investigate the corrosion products, EDX was performed at 40 μm at three spectrum pits Figure 9 were taken that are highly corroded. It was observed major alloying elements are Fe, Cr, Mo and electrolyte anions indicated under EDX analysis. Hence it was disclosed that under long term electrochemical analysis, no degradations occur in amorphous layer. Furthermore, the passive layer majorly consists of Fe, Mo and Cr oxide layers before the pitting initiate [39]. Here, from the above discussion and comparing our previous investigation on plasma spray, we concluded the fabrication technique and optimization of spraying parameters boost the chemo-mechanical properties of the coatings. The low porosity and strict inter-mechanical locking of sprayed alloy with substrate provide outstanding properties even for prolonged immersion and high-speed wear testing. These properties can be further increased by adding new alloying elements, which form more stable oxides than Cr, Mo and Fe.

## 3. Materials and Methods

This section illustrates the detailed overview of materials and the methods used during the art of experimentation. The novel composition of iron-based amorphous system FeMoCrYCB (composition shown in Table 3) was used as feedstock powder. The commercially available powder (SAM1651) was investigated for particle size analysis. The particle size analysis distribution test was performed by the Mastersizer 3000 laser diffraction particle size analyzer manufactured by Malvern Panalytical in the United Kingdom. Figure 1a reveals the distribution of the calculated particle ranges of 15–90 μm. The major portion of particles has a size range of 30–50 μm. Literature studies show that coatings developed by small particles have higher amorphous content and cooling rates during solidification [40]. The medium carbon steel AISI 1035 was employed as a substrate due to its superior strength, toughness, wear resistance properties, and abundant use in local industries.

The AISI 1035 steel substrate (composition shown in Table 4) was prepared for powder deposition by performing a series of steps like cleaned with acetone, dry air, and polished to avoid any corrosion by-products on the surface. The Fb-H01 high-pressure abrasive sand blaster manufactured by China was used at 0.35 MPa to increase the surface roughness. The amorphous powder was prepared by pre-heating at 120 °C for 1 h. Atmospheric plasma spray ultrasonic plasma spraying equipment SX-80, manufactured by Guangzhou Sanxin Metal S & T Co., Ltd., China, was used to coat the substrate. The modified parameters (shown in Table 5) from our previous study for plasma spray, argon gas at 0.75 psi and 120–130 Lmin^−1^, were used as essential gases, while hydrogen gas at 0.75 psi and 30–35 Lmin^−1^ was utilized as a minor gas. The chiller maintains a temperature of −16 °C continuously during operation. As the coating was fabricated, the samples were transformed into a specific shape via wire arc cutting so that length, width, and height remained 1 cm × 1 cm × 1 cm. The obtained samples were again prepared (grading and polishing to a mirror image) for further examinations.

A Tescan Mira high-resolution analytical scanning electron microscope (SEM) manufactured by Tescan China was used to analyze the microstructure of powder and coatings. The phase analysis of the obtained coating was investigated by the GNR explorer X-ray diffractometer manufactured by GNR optica Novara, Itlay. The theta range (below 90 and 0.01 step scan size) was set during experimentation. The investigation on SAM1651 under high temperature was performed by high-performance DSC Instruments, Mettler Toledo, manufactured by an analytical laboratory instrument manufacturing company, USA, to obtain the investigation on SAM1651 under high temperature. The given results were tested under parameters with the scanning rate of 10 °C/min and heating up to (0 °C–900 °C). The mechanical testing like micro-vickers hardness tests was done by a Micro Vickers hardness tester TH715, and tribometry properties were investigated using a Pin-on-disk tribometer manufactured by Rtec Instruments, San Jose, CA, USA.

With 3 mm-diameter diamond indenter, wear tests were conducted under ambient temperature circumstances with a load of 10 N and a sliding speed of 150 revolutions per minute (rmp). Electrochemical corrosion was evaluated by active potentiodynamic polarization testing with the Reference 600^TM^ potentiostat system manufactured by Gamry Instrument, Warminster, PA, USA. The sample preparation for corrosion was performed with JCD 908S 80 W Soldering Iron Kit after making the sample conductive. The coating surface was sanded with grits of 150, 200, 800, 1000, 1500, and 2000 coarse silicon carbide paper and afterwards cleaned to reflect face with an Al_2_O_3_ arrangement. Before every wear and corrosion experiment, this practice was performed for substrate and coated samples. Sodium chloride (concentration up to sea level) was used as an electrolyte. Experimental setup: the three-electrode system (working electrode, counter electrode, and reference electrode) was designed before experimentation. A mirror image sample was wrapped in the epoxy resin and a soldering iron kit was used to make solder on the backside of the sample to make it perfectly conductive. The samples were immersed in a saline environment of 3.5 wt % NaCl at 25 °C.

## 4. Conclusions

The effective amorphous layer of iron-based coatings was finished by plasma spray (PS). PS is one of the effective technique to fabricate Fe-based amorphous alloy coatings. The fabricated layer via PS remains amorphous during investigation for its thermal, mechanical, and electrochemical properties. The outstanding anti-wear property was obtained by employing a load of 10 N under dry sliding conditions. The amorphous layer produces a very low coefficient of friction compared to mild steel over high speed. In electrochemical potentiodynamic polarization, the amorphous layer produces an impressive passive layer of chromium, iron, and molybdenum before pitting initiation and protects the AISI 1035 against corrosion.

Additionally, the sample produces highly effective anti-corrosion resistance up to 48 h soaked in NaCl with around 200 μm of thickness. Here it is observed that strong interlocking-mechanical bonding increase the properties of coatings. Hence, it is confirmed that the use of the amorphous coating in our local industries can not only mitigate the corrosion and wear issues, but can also save billions of dollars for our economy. It is believed from the current experimentation that the effectivity of the SAM1651 alloy against the drastic conditions is highly notable and It can be increased further by the addition of new alloying elements as well as modifying the employed thermal technique.

## Figures and Tables

**Table 1:** Tribology and hardness of substrates and coated samples.

Samples	Hardness (Hv)	Force (N)	Wear Rate mm^3^N^−1^m^−1^
Amorphous	1140 ± 1%	10	0.0000000122124 (±2%)
AISI 1035	227 ± 1%	10	0.000000159716 (±2%)

**Table 2:** Electrochemical Corrosion Result.

Tested Samples and Hours	Corrosion Potential(V)	Corrosion Current Density	Beta Anode (V/Decade)	Beta Cathode (V/Decade)	Rate of Corrosion (mpy)
1 h Immersion PD-3	−1.0210325	1979.07 μA/cm^2^	0.6475 ± (0.01023)	−0.544 ± (0.00725)	9.25 × 10^−4^ (±1%)
24 h Immersion PD-1	−0.259503	2.25425 μA/cm^2^	0.08593 ± 7.9088 × 10^−4^	−0.20133 ± 0.00161	0.0343 × 10^−4^ (±1%)
48 h Immersion PD-2	−0.583870009	12.38734 μA/cm^2^	0.06737 ± 8.3756 × 10^−5^	−1.1648 ± 0.00785	1.88 × 10^−4^ (±1%)

**Table 3:** Chemical composition of Powder (wt %).

Elements	Fe	Mo	Cr	Y	C	B
wt %	48	14	15	2	15	6

**Table 4:** Composition of the 1035-Substrate (wt %).

Elements	C	*p*	Mn	S	Fe
wt %	0.350	0.040	0.90	0.050	98.66

**Table 5:** Parameters used for Atmospheric Plasma Spray.

Voltage	Spray Thickness	Current	Feeding Rate	Spray Distance	Power
50 V−60 V	0.2 mm	200–800 A	25 gmin^−1^	150 mm	40 KW

## Data Availability

Available on request and with regulations.

## Sample Availability

All Samples were used. Not Available at the moment.
